# Microbial Community Composition in Take-All Suppressive Soils

**DOI:** 10.3389/fmicb.2018.02198

**Published:** 2018-09-19

**Authors:** Paola Durán, Gonzalo Tortella, Sharon Viscardi, Patricio Javier Barra, Victor J. Carrión, María de la Luz Mora, María José Pozo

**Affiliations:** ^1^Scientific and Technological Bioresource Nucleus, Universidad de La Frontera, Temuco, Chile; ^2^Biocontrol Research Laboratory, Universidad de La Frontera, Temuco, Chile; ^3^Departamento de Procesos Diagnósticos y Evaluación, Facultad de Ciencias de la Salud, Universidad Católica de Temuco, Temuco, Chile; ^4^Department of Microbial Ecology, Netherlands Institute of Ecology (NIOO-KNAW), Wageningen, Netherlands; ^5^Department of Soil Microbiology and Symbiotic Systems, Estación Experimental del Zaidín (CSIC), Granada, Spain

**Keywords:** *Gaeumannomyces graminis*, microbial diversity, suppressive soils, take-all, real time PCR

## Abstract

*Gaeumannomyces graminis* var. *tritici* (Ggt) is the main soilborne factor that affects wheat production around the world. Recently we reported the occurrence of six suppressive soils in monoculture areas from indigenous “Mapuche” communities, and evidenced that the suppression relied on the biotic component of those soils. Here, we compare the rhizosphere and endosphere microbial community structure (total bacteria, actinomycetes, total fungi, and ascomycetes) of wheat plants grown in suppressive and conducive soils. Our results suggested that Ggt suppression could be mediated mostly by bacterial endophytes, rather than rhizosphere microorganisms, since the community structure was similar in all suppressive soils as compared with conducive. Interestingly, we found that despite the lower incidence of take-all disease in suppressive soils, the Ggt concentration in roots was not significantly reduced in all suppressive soils compared to those growing in conducive soil. Therefore, the disease suppression is not always related to a reduction of the pathogen biomass. Furthermore, we isolated endophytic bacteria from wheat roots growing in suppressive soils. Among them we identified *Serratia* spp. and *Enterobacter* spp. able to inhibit Ggt growth *in vitro*. Since the disease, but not always pathogen amount, was reduced in the suppressive soils, we propose that take all disease suppressiveness is not only related to direct antagonism to the pathogen.

## Introduction

Take-all disease is caused by *Gaeumannomyces graminis* (Sacc.) Arx et Olivier var. tritici (Walker) or Ggt. This fungus is an ascomycete belonging to the *Magnaporthe aceae* family, and it affects barley, rye, and other related grasses as triticale. However, it is best known for its notorious negative impact on wheat production (Cook, 2003). Southern Chile produces around 85% cereals, where 40% is wheat (ODEPA, 2018), and Ggt is the main soilborne factor causing crop loss (Moya-Elizondo et al., 2015). The fungus *G. graminis* can survive as saprophyte on infected or dead root and crown debris from previous crops causing primary infection through parasitism, thus infecting the next wheat crop (Hornby, 1983). Roots come into contact with the ascospores and dark runner hyphae of Ggt. The fungus colonize the surface, and then penetrates directly through hyaline hyphae beneath the hyphopodia into the roots cortex and across the endodermis into the stele, obtaining nutrients, carbon, and energy, hence triggering a secondary infection (Gilligan et al., 1994). Although the pathophysiology of the fungus is well known, its presence continues to be a problem due to the lack of effective methods for pathogen detection and control. Consequently, crop rotation remains a determinant cultural practice to diminish disease incidence. Therefore, the quantification of Ggt is crucial for future studies, in this sense, several efforts have focused on the development of an efficient molecular method to detect and quantify the presence and abundance of Ggt in soil or plant tissues. Early studies described a slot-blot hybridization technique for the semi-quantitative detection of Ggt in wheat roots and soils (Harvey and Ophel-Keller, 1996). Later, a quantitative DNA soil assay for Ggt detection was developed, but the resolution was low compared to the background signal (Herdina and Roget, 2000). Recently, the South Australian Research and Development Institute developed Predicta B^TM^, a series of tests for prediction and detection of many infectious plant diseases, including take-all (Ggt and *G. graminis* var. *avenae*, Gga). DNA from total *G. graminis* (Ggt and Gga) and from Gga alone was detected and quantified by real-time PCR (Ophel-Keller et al., 2008; Bithell et al., 2012). Ggt DNA was calculated by subtracting Gga DNA amount from the calculated *G. graminis* total DNA. However, this method is indirect and depends on multiple PCR reactions, significantly increasing the cost and time associated with the analysis.

Suppressiveness is defined as the ability of a natural soil to reduce or suppress the activity of plant pathogens, mostly due to the presence and activity of soil microorganisms. The presence of soil microorganisms increases the ecosystem resilience by creating redundancy in ecosystem services, making soil less vulnerable to short-term changes in the environment (Wall et al., 2012). Several studies have shown that conducive soils, where the incidence of take-all disease is elevated (Chng et al., 2015), can become suppressive under certain conditions: monoculture of susceptible host, Ggt presence, and take-all disease outbreak (Weller et al., 2002). Thus, suppressive soils are defined as soils where disease development is minimal despite the presence of an infective pathogen and a susceptible plant host (Mazzola, 2002). Despite that suppressive soils have been known for over 100 years and have been studied for more than five decades, they remain poorly understood (Chandrashekara et al., 2012; Löbmann et al., 2016). Considering the great potential that suppressive soils offer for sustainable pest management and plant biocontrol, the development of tools for their study and the understanding of the mechanisms leading to pathogen suppression should be a priority line of research.

In a previous study from our group, we characterized several suppressive soils and evidenced the essential role of soil microbial communities in the Ggt suppression by six soils managed under ancestral agronomic practices as monoculture for more than 10 years (Durán et al., 2017). However, there is little known regarding the structure of the rhizosphere microbial communities and pathogen abundance required to fully understand the role of different microbial groups in the control of Ggt in suppressive soils.

Considering the unique niche that suppressive soils offer in terms of harboring specialized microbial communities to suppress Ggt, the economic and biological importance of take-all disease in wheat production, and the current technical and economical limitations for its detection, the main goals of this study were: (i) to design specific primers as a tool to evaluate by qPCR Ggt abundance in soil and plant samples in supressiveness studies, (ii) To determine the structure of the rhizosphere and endosphere microbial community (total bacteria, actinomycetes, total fungi, and ascomycete group) harbored in suppressive soils, and (iii) targeted isolation of key microorganisms putatively involved in take-all disease suppression.

## Materials and Methods

### Selected Suppressive and Conductive Soils

Three volcanic soils previously identified by Durán et al. (2017) as suppressive (soils 2, 4, and 13) and one as conductive soil (soil 1, conducive control) were selected for greenhouse experiments (**Table 1A**). All suppressive soils have a large history of wheat monoculture for more than 10 years.

**Table 1A:** Chemical properties of rhizosphere soils used in this study (*n* = 3).

Soil	P (mg kg^-1^)	K (cmol+ kg^-1^)	pH (H_2_O)	OM (%)	Al sat^†^ (%)	CICE (cmol+ kg^-1^)	ΣΣ basis (cmol_(+)_kg^-1^)
1	60.3 ± 0.8^*a*∗^	2.8 ± 0.1^*a*^	5.9 ± 0.1^*a*^	12.2 ± 0.1^*d*^	0.6 ± 0.0^*c*^	16.9 ± 0.0^*a*^	16.9 ± 0.0^*a*^
2	41.5 ± 0.9^*cd*^	1.2 ± 0.0^*b*^	5.3 ± 0.0^*b*^	10.9 ± 0.2^*d*^	9.9 ± 0.1^*a*^	11.0 ± 0.1^*c*^	9.9 ± 0.1^*c*^
4	59.8 ± 0.2^*a*^	0.6 ± 0.0^*d*^	5.7 ± 0.0^*ab*^	15.1 ± 0.1^*a*^	1.1 ± 0.0^*b*^	9.4 ± 0.0^*d*^	9.3 ± 0.0^*c*^
13	5.6 ± 0.4^*k*^	0.9 ± 0.0^*c*^	5.6 ± 0.0^*c*^	13.5 ± 0.2^*b*^	0.5 ± 0.0^*c*^	15.7 ± 0.1^*b*^	15.6 ± 0.1^*b*^


*^†^Calculated as Al/cation exchange capacity [ΣΣ (K, Ca, Mg, Na, and Al)] × 100, *n* =3.*

*^∗^Different letters in the same column denote significant differences (Tukey’s test, *P* ≤ 0.05).*

Soil chemical analyses were determined as described in Durán et al. (2017): available phosphorus P (P-Olsen) was extracted by using 0.5 M NaHCO_3_ and analyzed with the molybdate method (Murphy and Riley, 1962). Organic matter (OM) was calculated by wet digestion (Walkley and Black, 1934). Soil pH was measured in 1:2.5 soil/deionized water suspensions. Exchangeable potassium (K^+^), calcium (Ca^2+^), magnesium (Mg^2+^), and sodium (Na^+^) were extracted with 1 M ammonium acetate (CH_3_COONH_4_) at pH 7.0 and analyzed by flame atomic adsorption spectrophotometry.

### Preparation of Ggt Inoculum and Media

For inoculum preparation (powder inoculum), oat kernels were soaked in water for 24 h and sterilized for three consecutive days at 121°C for 15 min. Then, a mycelial plug of Ggt strain KY689233 was inoculated onto sterile oats and incubated at room temperature for 20 days. Colonized oat kernels were blended, sieved to a particle size of 0.5–1.0 mm, and stored at 4°C until usage (Durán et al., 2017).

Fungi used as negative controls for the Ggt specificity test were routinely grown on Potato Dextrose Agar (PDA, OXOID) for 7 days at 25°C.

### Greenhouse Experiments

#### Experiment 1

Plastic containers containing 200 g of the selected soils (described in 2.1) were used in quintuplicate. Wheat seeds Otto cv were surface sterilized (15% ethanol plus 1% sodium hypochlorite for 2 min) and 5 seeds were used for each treatment. Ggt inoculum (powder inoculum) was applied at 0.1% in relation to soil weight (2 g), and all treatments were also performed in soil without Ggt inocula as controls (Durán et al., 2017). Plants were watered every 3 days, and Taylor and Foyd nutrient solution (Taylor and Foyd, 1985) was applied each 15 days.

#### Experiment 2

To standardize (or set up/test) the Ggt quantification method in wheat plants a new greenhouse experiment was performed in a soil collected in Piedras Negras from Southern Chile (**Table 1B**) following the conditions described for greenhouse experiment 1. Soils were inoculated with 0.1, 5, and 10% of powder Ggt inoculum, and non-infected soils were used as a control. Seedlings were watered every 3 days and fertilized with 50 mL of Taylor and Foyd nutrient solution every 15 days.

**Table 1B:** Chemical properties of Piedras negras (PN) soil used for qPCR validation for Ggt quantification.

Soil	P (mg kg^-1^)	K (cmol+ kg^-1^)	pH (H_2_O)	OM (%)	Al sat^†^ (%)	CICE (cmol+ kg^-1^)	ΣΣ basis (cmol_(+)_kg^-1^)
PN^∗∗^	9.5 ± 0.85	0.32 ± 0.00	5.75 ± 0.65	29 ± 1.80	0.174 ± 0.00	11.53 ± 0.01	11.47 ± 0.88


#### Sample Processing and DNA Extraction

After 40 days, plants were carefully harvested. Soil adhered to roots was considered as rhizosphere soil (the portion of the soil influenced by roots exudates) and for the endosphere, roots were surface-sterilized by repeated immersion in 80% v/v ethanol for 5 min and 4% v/v NaOCl for 20 min and rinsed three times with sterile distilled water (Shimizu, 2011) and reserved. The efficacy of tissue surface sterilization was confirmed by spreading 100 μl of the last rinsing step water in LB and PDA. DNA from endosphere and rhizosphere soils was extracted in three replicates using Power Soil DNA Isolation Kit (QIAGEN, United States), according to the manufacturer’s instructions. DNA was quantified and its purity evaluated using the A260/A280 and A260/A230 ratios provided by Multiskan^TM^ GO software.

### Analysis of Microbial Community Composition

The microbial community composition in most representative suppressive soils compared to the conductive sample was evaluated by DGGE using universal primer sets for total bacteria, actinomycetes, fungi, and ascomycetes. Primers used for each microbial group are shown in **Table 2A**. The quality and quantity of the resulting amplicons for all DDGE were assessed by electrophoresis in a 1.5% agarose gel and compared to 100 bp DNA mass ladder bands (Invitrogen). The primer set generates amplicons between 300–500 bp. The DGGE analysis was performed using a DCode system (Bio-Rad Laboratories, Inc.). Twenty-five μL PCR product were loaded onto 6% (w/v) polyacrylamide gel with 40–70% gradient (urea and formamide). The electrophoresis was run for 16 h at 75 V. The gel was then stained with SYBR Gold (Molecular Probes, Invitrogen Co.) for 30 min and photographed on an UV transilluminator. The DGGE banding profiles Clustering as dendrogram was carried out by using Phoretix 1D analysis software (Clarke, 1993) (TotalLab Ltd., United Kingdom). The correlation between bacterial communities (biological parameters) and chemical soil properties (ecological parameters) was visualized by distance based redundancy analysis (dbRDA) by using Primer 7+ Permanova software (Primer-E Ltd., Ivybridge, United Kingdom; Clarke, 1993). The *in silico* analysis was also used to estimate bacterial diversity by richness (S), Shannon–Wiener index (H′), and dominance by Simpson Index (D) represented by 1- D or 1-λ (Sagar and Sharma, 2012).

**Table 2A:** Sequence of primers used in DGGE analyses.

Microbial group	Primer set	Sequence	Reference
Total bacteria	EUBf933-GC EUBr1387	5′-GCA CAA GCG GTG GAG CAT GTG G-3′ 5′-GCC CGG GAA CGT ATT CAC CG-3′	Iwamoto et al., 2010
Actinobacteria	f243 r513-GC	5′-GGA TGA GCCCGCGGCCTA-3′ 5′-gc-CGG CCG CGG CTG CTG GCA CGTA-3′, GC-clamp: CGC CCG CCG CGC GCG GCG GGC GGG GCG GGG GCA CGG GGG G	Heuer et al., 1997
Total fungi	fNS1 rNS8 NS7-GC f1r	5′-GTA GTC ATA TGC TTG TCT C-3′ 5′-TCC GCA GGT TCA CCT ACG GA-3′ 5′-GAG GCA ATA ACA GGT CTG TGA TGC-3, GC-clamp: CGC CCG GGG CGC GCC CCG GGC GGG GCG GGG GCA CGG GGG 5′-CTT TTA CTT CCT CTA AAT GAC C-3′	Cea et al., 2010
Ascomycete	ITS4asco ITS3-GC	5′-CGT TAC TRR GGC AAT CCC TGT TG-3′ 5′-gc-GCA TCG ATG AAG AAC GCA GC-3′, GC-clamp: CGC CCG CCG CGC GCG GCG GGC GGG GCG GGG GCA CGG GGGG	Nikolcheva and Bärlocher, 2004; White et al., 1990


### *Gaeumannomyces graminis* var. *tritici* Quantification

#### Primer Design and Validation

Oligonucleotide primer set to target internal transcribed spacer (ITS2) ribosomal region sequences from *G. graminis* var. *tritici* were designed using Primer3 software^1^. Primer specificity was tested *in silico* by a similarity search against DNA sequences in ITS2 database (Merget et al., 2012). The specificity of the Ggt sequences *in silico* was verified in alignments using Clustal Omega^2^, and a phylogenetic tree, which shows the affiliation of Ggt in relation to others amplified strains, was constructed using MEGA 7 software (Molecular Evolutionary Genetics Analysis version 7.0 for bigger datasets) (Kumar et al., 2016).

The specificity of the designed primers was evaluated against several fungal species including, *Gaeumannomyces graminis* var. *tritici* (Ggt strain KY689233), as positive control; and *Aspergillus niger*, *Mortierella* sp., *Rhizopus* sp., *Thelebolus* sp., *Pseudogymnoascus* sp., *Cosmospora* sp., *Lecanicillium* sp., *Alternaria* sp., and *Diaporthe* sp. as negative control and roots and rhizosphere soils of wheat plants both infected and non- infected (negative control). Conventional PCR with the selected primer sets was performed as follow: 10 min at 95°C followed by 35 amplification cycles of 1 min at 95°C, 1 min annealing at 58.4°C, and 1 min elongation at 72°C, with a final extension of 7 min at 72°C.

#### Real Time PCR to Validate the Primer Design

Quantitative PCR was performed in an Applied Biosystems Step One^TM^ Real-Time PCR System in 12 μl reaction mixtures, containing BrillantII^®^SYBR^®^, Green real-time PCR master mix (Stratagene, Agilent Technologies Company, United States), 1 μl of 1:10 Ggt DNA dilution (to determine standard curve), 1 μL sample DNA, and 600 nM of each primer. Real-time PCR was performed in triplicate under the described conditions: an initial denaturing step at 95°C for 10 min and 35 cycles at 95°C for 15 s, 58.4°C for 20 s, and 72°C for 40 s. The specificity of amplified products was checked immediately after the amplification process by analyzing the dissociation curves generated from 60 to 95°C at 0.3°C intervals. Cycle threshold values (*C*_T_) were converted to picograms of DNA per gram of sample (root or rhizospheric soil) using a reference standard curve made of 10-fold dilutions for DNA at 0.8 to 8 × 10^-7^ ng DNA from Ggt isolate. Additionally, the amplification specificity was checked by sequencing the PCR products at Macrogen Inc., Korea. Sequences were deposited in the GenBank database (soil: MG754063, rhizosphere: MG754064, roots: MG893091).

To determine the copy number of Ggt genomes in the samples, we used the following formula:

$$\frac{DNA\,\;G\,\;gt\,\;sample{\left(\frac{ng}{\mu L}\right)}^{*}1\times 10^{-9}}{m\,(g)genome}/13$$

where 13 represents the number of copies of the amplified fragment in the Ggt genome, and:

$$\frac{G\,\;gt\,\;genome\,\;weight\left(43.768.664\,\;bp\right)\times average\,\;MW\,\;double\left(660\frac{g}{mol}\right)}{n\overset{\circ }{-}avogadros}$$

#### Quantification of Ggt by Real Time Quantitative PCR (qPCR)

For standardization of Ggt quantification, tenfold serial dilutions of Ggt genomic DNA from 0.8 to 8 × 10^-5^ ng μL^-1^ (obtained from Ggt pure culture in PDA), were prepared in triplicate and used for real-time PCR analysis. Abundance analysis of Ggt was performed in an Applied Biosystems Step One^TM^ Real-Time PCR System in 12 μl reaction mixtures, containing Brillant^®^II SYBR^®^, Green QPCR master mix (Stratagene, Agilent Technologies Company, United States), 1 μl 1:10 Ggt DNA dilution (to determine standard curve) and 1 μL sample DNA and 600 nM of each primer. The specific selected primers GGT2F/GGT168R were used. PCR was performed in triplicate under the conditions described in 2.7.2

### Endophytic Bacteria Isolation From Wheat Plants Grown in Suppressive Soils

Given the importance of endophytic bacteria strains denoted in this study, roots and shoots from wheat plants growing in the different soils were separated, surface sterilized by repeatedly immersing the samples in 80% v/v ethanol for 5 min and 4% v/v NaOCl for 20 min, and then rinsed three times with sterile distilled water. Tissue samples were macerated and homogenized in 1 mL sterile saline solution (0.85 % v/v NaCl). One hundred microliters of homogenized tissue dilutions were spread onto the general media Luria-Bertani (LB) and incubated at 30°C for 2 days. The efficacy of tissue surface sterilization was confirmed by spreading 100 μl of the last rinsing step water in LB (Durán et al., 2014).

Endophytic bacteria isolated from suppressive soils were tested for *in vitro* antagonistic activity against *G. graminis* var. *tritici*. Briefly, Ggt was grown on PDA plates at 25°C for 1 week. Agar disks (4-mm diameter) containing Ggt were aseptically incised and transferred to the center of agar plates containing fresh LB/PDA (1:1) media. Then, two drops (5 μL) of previously isolated endophytic bacteria suspension were taken from overnight LB cultures (washed three times with NaCl buffer) and placed on two diametric positions at 2 cm from the agar disk containing the Ggt inoculum. Fungal mycelia growth was measured after 3, 5, and 7 days of incubation at 25°C in the darkness as previously described (Durán et al., 2014).

### Identification of Endophytic Selected Strains

Genetic characterization of selected bacteria was based on partial sequencing of 16S rRNA gene. The 16S rRNA gene fragments were amplified by PCR with universal bacterial primers set 27f and 1492r (Peace and Stills, 1994). After starting at 94°C for 5 min, PCR amplification was carried out for 35 cycles at 94°C for 1 min, 52°C for 1 min, and 72°C for 2 min. The PCR products were purified and sequenced by Macrogen Inc., (Korea). Sequences were deposited under accession nos. MF623050, MF623051, and MF623052, and then compared with those in the GenBank database.

### Statistical Analyses

For the microbial community composition, data normality was analyzed according to Kolmogorov’s test. The similarity between bacterial communities was visualized in Distance based redundancy analyses (dbRDA), by using Primer 7 software (Primer-E Ltd., Ivybridge, United Kingdom), which showed a Bray–Curtis similarity index higher than 60% and less than 0.14 stress values (Clarke, 1993). Values were given as means ± standard errors. Differences were considered significant when the *P*-value was lower than or equal to 0.01.

For the qPCR analysis, the *C*_T_ value for each real-time PCR reaction was calculated using the Step One plus^TM^ Real-Time PCR System (v 2.3). Then, to compare between different DNA dilution series generated from different treatments, the logarithm of the linear regressions of known concentrations of the Ggt target DNA versus the *C*_T_ values were calculated for each DNA standard curve using SPSS software (SPSS Inc. v. 20). Standard regression lines chosen as reference curves were used for transforming the experimental *C*_T_ values into copy numbers of target DNA in Ggt, inocula, roots, and rhizosphere soils. The PCR efficiency (E) was calculated from the slope of the standard curve using the equation E = 10^[-1/*slope*]^. The equivalence of slopes and intercepts of standard regression lines were tested using analysis of variance (ANOVA) in SPSS software.

The results obtained in the *in vitro* assay were analyzed by a one-way analysis of variance (ANOVA) and compared by Tukey test, using SPSS software (SPSS, Inc.).

## Results

### Microbial Community Composition of Different Microbiological Groups (Total Bacteria, Actinomycetes, Total Fungi, and Ascomycetes)

**FIGURE 1:**
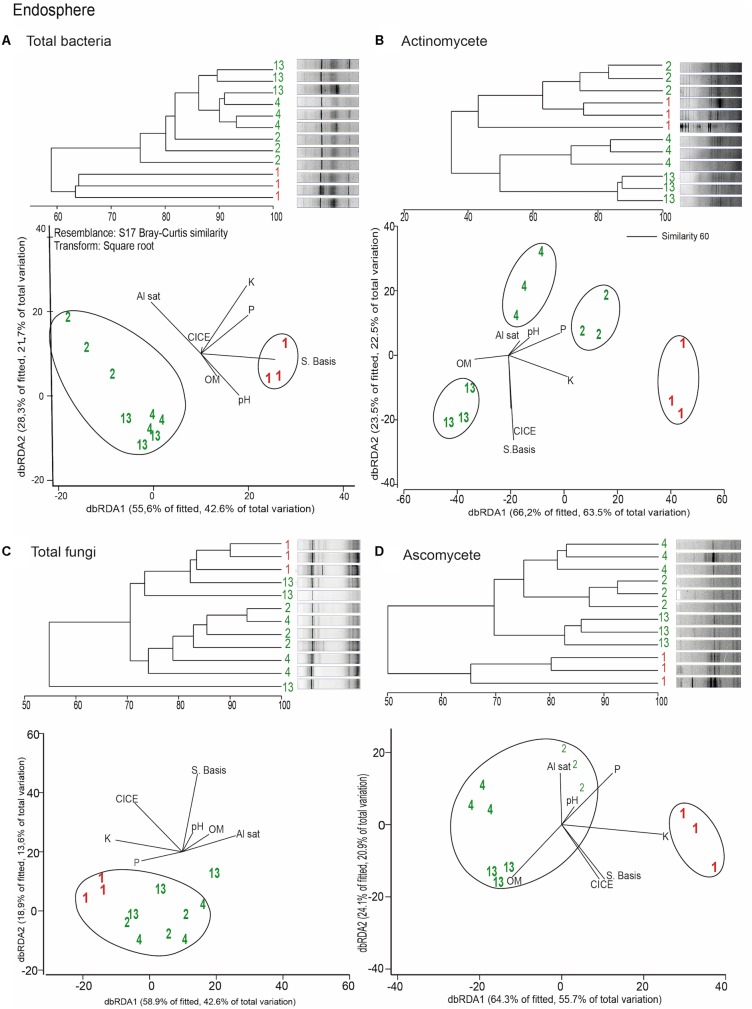
Dendrograms and distance based redundancy analyses (dbRDA) plots of the endosphere of wheat plants grown in conducive and suppressive soils, based on DGGE profiles of total bacteria **(A)**, actinomycetes **(B)**, total fungi **(C)**, and ascomycete **(D)** and soil chemical parameters (P, K, OM, Al sat, CICE, and Σ basis). Soil parameters are represented with black lines in the dbRDA plots, the length and position represent the correlation (*P* < 0.05) with the endosphere microbial community composition. Red and green letters represent the endophytic community of wheat plants grown in conductive or suppressive soils, respectively.

**FIGURE 2:**
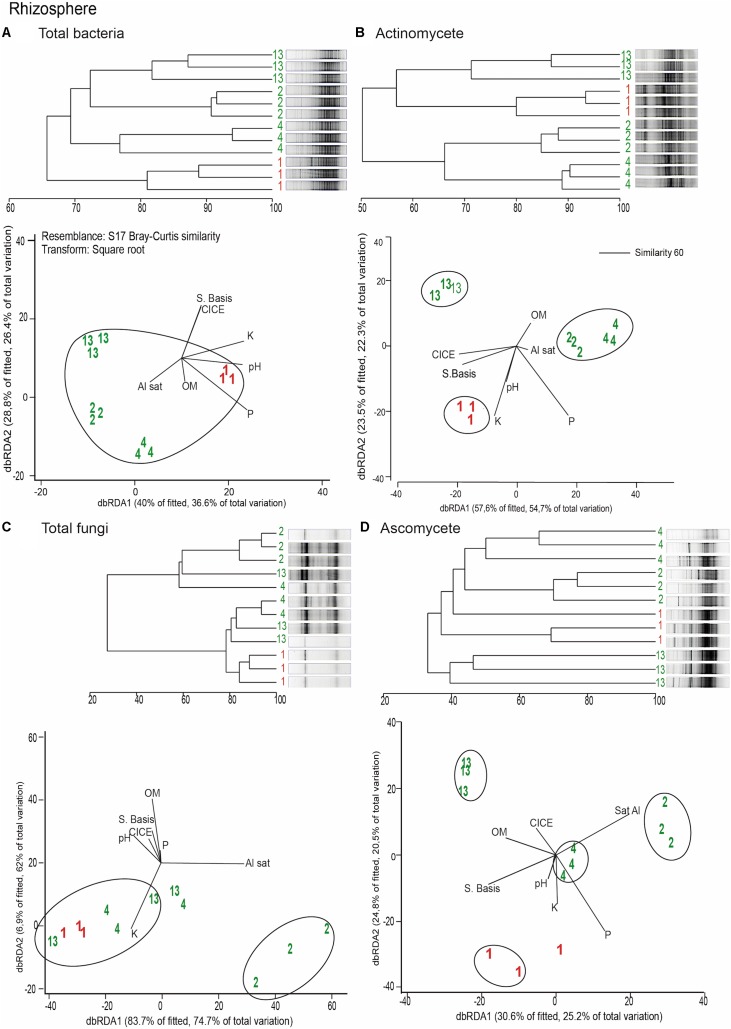
Dendrograms and distance based redundance analyses (dbRDA) plots of the rhizosphere of wheat plants grown in conducive and suppressive soils, based on DGGE profiles of total bacteria **(A)**, actinomycetes **(B)**, total fungi **(C),** and ascomycete **(D)** and soil chemical parameters (P, K, OM, Al sat, CICE, and Σ basis). Soil parameters are represented with black lines in the dbRDA plots, the length and position represent the correlation (*P* < 0.05) with the endosphere microbial community composition. Red and green letters represent the endophytic community of wheat plants grown in conductive or suppressive soils, respectively.

**FIGURE 3:**
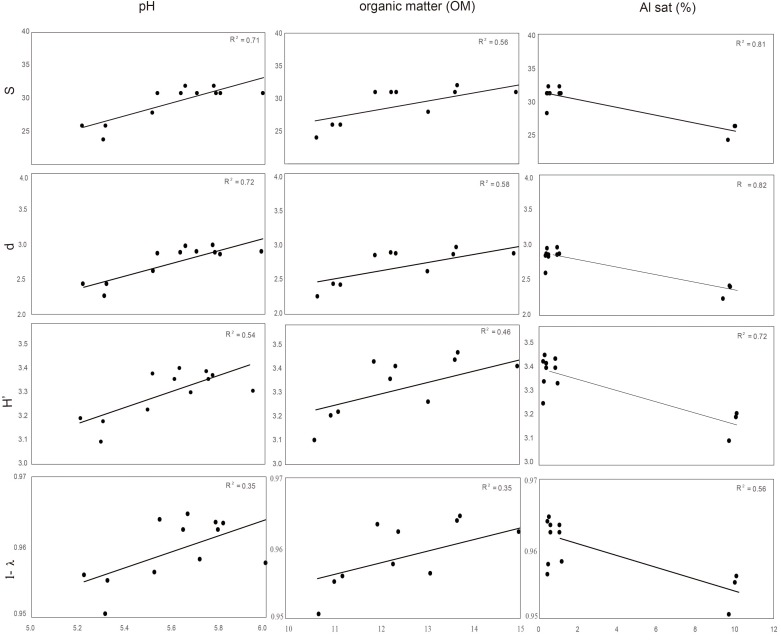
Correlation between biodiversity index S (species), d (individual), H′ (Shannon), and Simpson (expressed as 1- λ) of total bacteria rhizosphere, and soil parameters (pH, OM, and Sat Al).

Distance based redundancy analyses (dbRDA) of the microbial communities based on the DGGE profiles of total bacteria, actinomycetes, total fungi and ascomycetes of the endosphere and rhizosphere of wheat plants grown in conducive and suppressive soils is shown in **Figures 1**, **2**, respectively. In the endosphere, the results showed that at 60% similarity total bacteria was the main variable explaining the separation between endosphere of wheat plants grown in suppressive and conducive soils (**Figure 1A**). A similar trend was observed in the ascomycetes group (**Figure 1D**), where at 60% of similarity both treatments, endosphere from plants grown is suppressive and conducive soils, were clustered in different groups. Regarding to the actinomycetes in the endosphere samples, all treatments clustered independently in different groups (**Figure 1B**) whereas no separation was observed regarding the total fungal community in the endosphere (**Figure 1C**). In rhizosphere soils, no separation between suppressive and conducive control soil was evidenced, where the community structure was mostly influenced by chemical parameters, mainly P, Al sat, pH, OM (**Figures 2A–D**). *In silico* analysis was also used to estimate bacterial diversity by richness expressed as follow: number of individuals (N), number of species (S), Shannon–Wiener index (H′) to estimate diversity by abundance, and Simpson Index (D) represented by 1- D or 1-λ to estimate diversity by dominance. Thus, considering the main soil factors that influence the chemical parameters of andisol soils, as pH, OM, and Al Sat, we found that all biodiversity indexes were positively correlated with pH and OM, and negatively correlated with Al sat in rhizosphere soil (**Figure 3** and **Supplementary Table 1**).

We found that despite an elevated diversity, expressed in number of individuals (N from 325 to 540 in endosphere, and from 30,000 to 35,000 in rhizosphere), and lower dominance in the case of endophytic bacteria (Simpson 0.74–0.85) with respect to rhizosphere soil (Simpson >0.95), no significant differences were found between suppressive and conducive soils in both endosphere and rhizosphere microorganisms (**Table 3**). The actinomycete group showed major diversity (S, N, d, H′) and dominance (**1-λ**) in suppressive soils in the rhizosphere, but not in the endosphere. In contrast, ascomycete showed less diversity in the endosphere of suppressive soils but not in rhizosphere soil. Finality, no significant differences between suppressive and conducive soils in both endosphere and rhizosphere of total fungi were found.

### Correlation Between Endophytic Ascomycete and Other Microbial Groups

**FIGURE 4:**
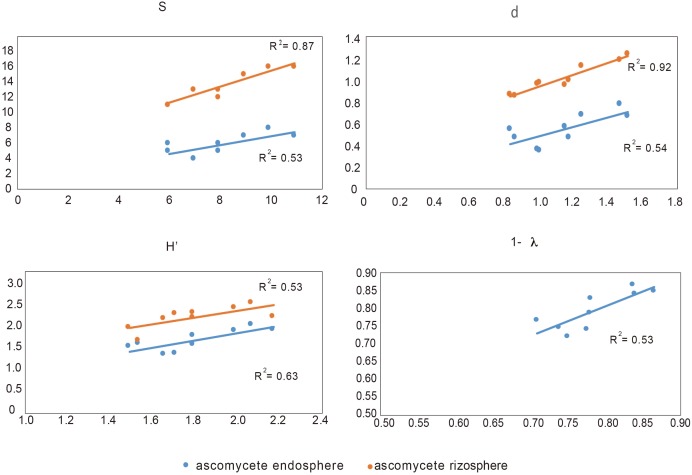
Correlation between biodiversity index S (species), d (individual), H′ (Shannon), and Simpson (expressed as 1- λ) of endophytic bacteria and rhizosphere and endosphere ascomycetes.

In order to determine the influence of the ascomycete group (to which the pathogen belongs) on the rest of microbial groups, we analyzed the Pearson correlation between them and their respective Shannon index (see **Supplementary Table 2**). We found that the ascomycete, both rhizosphere and endosphere in suppressive soils, are correlated with endophytic bacteria, whereas endophytic ascomycetes from conducive are not related with any other microbial groups. In this context, and considering all biodiversity indexes, we found that endophytic bacteria are related with both ascomycete group (rhizosphere and endosphere), except in the Simpson index (1- λ) that it was only related with endosphere ascomycete (**Figure 4**).

### Primers Design and Specificity

The *in silico* primer design generated seven hundred primer combinations able to amplify a region of the Ggt KY689233 sequence. However, only twenty-four combinations (2 forward and twelve reverse oligonucleotides, **Table 2B**) recognized *G. graminis* var. *tritici* as a target in the ITS2 database. The forward primer GGT2F (5′ AGCCCAGCTTGGTGTTGG 3′) was selected for optimization of the real-time PCR because of its higher *T*_m_ (58.4°C) compared to the other forward primer (56.1°C). Twelve primer combinations were tested by real-time PCR, but only two pairs, GGT2F with GGT200R (5′-GAACGAAGCGCGTTTTACC-3′), and GGT2F with GGT168R (5′ CGTTTTACCGCGAGTTACTG 3′), provided a suitable standard curve, with efficiencies of 95 and 98%, respectively. Given that the primer combination GGT2F with GGT168R had a greater efficiency, this pair of primers was selected for further experiments.

**Table 2B:** Designed primers for Ggt quantification.

	Oligo	Sequence (5′ - 3′)	*T*_m_	GC%
1	GGT1F	AAGCCCAGCTTGGTGTTG	56.1	55.56
2	GGT2F	AGCCCAGCTTGGTGTTGG	58.4	61.11
3	GGT148R	GCGAGTTACTGCGTTCAG	59.5	57.89
4	GGT168R	CGTTTTACCGCGAGTTACTG	58.4	50.00
5	GGT218R	CGCGAGTTACTGCGTTCAG	59.5	57.89
6	GGT220R	GAACGAAGCGCGTTTTACC	57.3	52.63
7	GGT307R	CGCGAGTTACTGCGTTCA	56.1	55.56
8	GGT323R	ACCGCGAGTTACTGCGTT	59.6	57.89
9	GGT350R	GCGTTTTACCGCGAGTTAC	57.3	52.63
10	GGT351R	CCGCGAGTTACTGCGTTC	58.4	61.11
11	GGT444R	AACGAAGCGCGTTTTACC	53.9	50.00
12	GGT595R	CGCGAGTTACTGCGTTCAG	59.5	57.89
13	GGT599R	GAACGAAGCGCGTTTTACC	57.3	52.63
14	GGT682R	ACCGCGAGTTACTGCGTTC	59.5	57.89


**Table 3:** Biodiversity indexes for different microbial groups (Data in the same column not sharing a letter in common are significantly different according to Tukey test, *p* < 0.05).

	Biodiversity index					
	
Sample	S	N	d	J	H′	1-λ
**Total bacteria (endosphere)**					
S1	8.67 ± 0.88^ab^	526 ± 77.42^a^	1.22 ± 0.12^ab^	0.91 ± 0.01^a^	1.96 ± 0.12^a^	0.83 ± 0.02^ab^
S2	10.0 ± 0.58^a^	538 ± 71.90^a^	1.44 ± 0.08^a^	0.91 ± 0.00^a^	2.10 ± 0.05^a^	0.85 ± 0.01^a^
S4	7.67 ± 0.33^ab^	366 ± 10.71^a^	1.13 ± 0.06^ab^	0.88 ± 0.01^a^	1.78 ± 0.03^ab^	0.78 ± 0.00^bc^
S13	6.33 ± 0.33^b^	327 ± 20.03^a^	0.92 ± 0.05^b^	0.86 ± 0.01^a^	1.58 ± 0.05^b^	0.74 ± 0.01^c^
**Total bacteria (rhizosphere)**					
S1	31 ± 0.00^a^	34998 ± 1362^a^	2.87 ± 0.01^a^	0.97 ± 0.01^a^	3.34 ± 0.02^a^	0.96 ± 0.00^a^
S2	25 ± 0.67^b^	29980 ± 819^a^	2.36 ± 0.06^b^	0.97 ± 0.00^a^	3.15 ± 0.03^b^	0.95 ± 0.00^a^
S4	31 ± 0.33^a^	33748 ± 682^a^	2.91 ± 0.03^a^	0.97 ± 0.01^a^	3.34 ± 0.03^a^	0.96 ± 0.00^a^
S13	30 ± 1.20^a^	33878 ± 1495^a^	2.81 ± 0.11^a^	0.98 ± 0.00^a^	3.33 ± 0.05^a^	0.96 ± 0.00^a^
**Actinomycete (endosphere)**					
S1	9.33 ± 0.67^a^	10889 ± 1265^a^	0.90 ± 0.08^a^	0.96 ± 0.00^b^	2.14 ± 0.07^a^	0.87 ± 0.01^a^
S2	10.0 ± 1.00^a^	7332 ± 840^ab^	1.01 ± 0.10^a^	0.99 ± 0.00^a^	2.27 ± 0.09^a^	0.89 ± 0.01^a^
S4	8.00 ± 0.58^a^	4258 ± 446^b^	0.84 ± 0.06^a^	0.98 ± 0.01^a^	2.04 ± 0.08^a^	0.86 ± 0.01^a^
S13	8.33 ± 0.33^a^	5490 ± 241^b^	0.85 ± 0.03^a^	0.99 ± 0.00^a^	2.10 ± 0.04^a^	0.88 ± 0.01^a^
**Actinomycete (rhizosphere)**					
S1	15.3 ± 0.33^b^	22305 ± 252^a^	1.43 ± 0.03^b^	0.97 ± 0.00^a^	2.64 ± 0.03^b^	0.92 ± 0.00^b^
S2	20.0 ± 0.00^a^	25620 ± 1528^a^	1.87 ± 0.01^a^	0.98 ± 0.00^a^	2.94 ± 0.01^a^	0.95 ± 0.00^a^
S4	21.7 ± 0.33^a^	26579 ± 677^a^	2.03 ± 0.03^a^	0.97 ± 0.00^a^	2.99 ± 0.03^a^	0.95 ± 0.00^a^
S13	21.0 ± 1.15^a^	22624 ± 1019^a^	1.99 ± 0.11^a^	0.96 ± 0.01^a^	2.91 ± 0.09^a^	0.94 ± 0.01^ab^
**Total fungi (endosphere)**					
S1	7.67 ± 0.67^a^	11905 ± 720^a^	0.70 ± 0.06^a^	0.91 ± 0.01^a^	1.86 ± 0.07^a^	0.82 ± 0.01^a^
S2	9.67 ± 1.33^a^	13257 ± 1446^a^	0.91 ± 0.13^a^	0.91 ± 0.01^a^	2.04 ± 0.14^a^	0.84 ± 0.02^a^
S4	9.67 ± 1.20^a^	12729 ± 2213^a^	0.92 ± 0.11^a^	0.91 ± 0.01^a^	2.06 ± 0.12^a^	0.84 ± 0.02^a^
S13	9.00 ± 0.58^a^	11884 ± 3230^a^	0.86 ± 0.04^a^	0.91 ± 0.02^a^	2.01 ± 0.10^a^	0.83 ± 0.02^a^
**Total fungi (rhizosphere)**					
S1	13.7 ± 0.33^a^	22194 ± 1140^a^	1.27 ± 0.03^a^	0.90 ± 0.01^b^	2.35 ± 0.04^a^	0.88 ± 0.01^a^
S2	3.00 ± 0.00^b^	5317 ± 616^b^	0.23 ± 0.00^b^	0.99 ± 0.00^a^	1.08 ± 0.00^a^	0.66 ± 0.00^a^
S4	8.33 ± 3.18^a^	14588 ± 6196^ab^	0.75 ± 0.31^ab^	0.93 ± 0.00^b^	1.72 ± 0.54^a^	0.74 ± 0.14^a^
S13	8.67 ± 1.33^a^	12753 ± 3657^ab^	0.81 ± 0.12^ab^	0.89 ± 0.02^b^	1.90 ± 0.18^a^	0.82 ± 0.04^a^
**Ascomycete (endosphere)**					
S1	9.33 ± 0.33^a^	10342 ± 1484^a^	0.90 ± 0.04^a^	0.97 ± 0.01^a^	2.16 ± 0.04^a^	0.88 ± 0.01^a^
S2	7.33 ± 0.33^a^	6080 ± 375^ab^	0.73 ± 0.03^a^	0.99 ± 0.00^a^	1.96 ± 0.05^a^	0.86 ± 0.01^ab^
S4	5.00 ± 0.58^b^	3941 ± 693^b^	0.48 ± 0.06^b^	0.99 ± 0.00^a^	1.58 ± 0.12^b^	0.79 ± 0.03^bc^
S13	5.00 ± 0.58^b^	4960 ± 1095^b^	0.47 ± 0.06^b^	0.94 ± 0.02^a^	1.49 ± 0.08^b^	0.75 ± 0.01^c^
**Ascomycete (rhizosphere)**					
S1	11.33 ± 0.67^b^	159073 ± 10539^a^	0.86 ± 0.06^b^	0.87 ± 0.03^a^	2.11 ± 0.08^ab^	0.86 ± 0.01^a^
S2	15.67 ± 0.33^a^	194864 ± 27794^a^	1.21 ± 0.03^a^	0.88 ± 0.04^a^	2.41 ± 0.09^a^	0.89 ± 0.01^a^
S4	12.67 ± 0.33^b^	135092 ± 33071^a^	0.99 ± 0.01^b^	0.90 ± 0.00^a^	2.28 ± 0.03^ab^	0.89 ± 0.00^a^
S13	11.67 ± 0.67^b^	112592 ± 25021^a^	0.92 ± 0.04^b^	0.79 ± 0.05^a^	1.95 ± 0.15^b^	0.81 ± 0.04^a^


*S, total species; N, total individual; d, Mergalef index; J, Pielou’s index; H′, Shannon index; 1-λ, Simpson index.*

**FIGURE 5:**
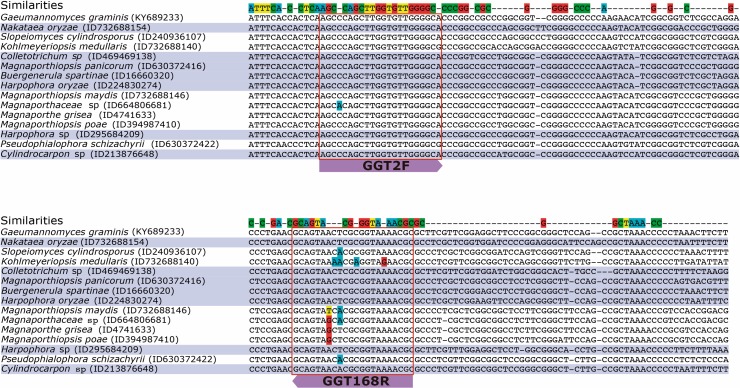
Multiple sequence alignment of the ITS region of the strains which the selected primer pair could amplify. The strain accession numbers are in parentheses.

The *in silico* results showed that the primer set GGT2F/GGT168R could amplify seventy-three species belonging to twenty-six different Genera, which could interfere in the quantification of Ggt. However, only fifteen of these species amplified in the same *T*_m_ (58°C), and eight species showed high identity when the primers were aligned to the ITS sequence (**Figure 5**). From these eight species, two belonged to *Harpophora* spp., which is a soilborne and apparently seedborne fungus; based on phylogenetic analyses, *Harpophora* spp. has been reported to be related to the root-infecting species in the genus *Gaeumannomyces* (Luo et al., 2015; Zhang et al., 2016). The other five species belonged to five different genera and three families: Glomerellaceae, Nectriaceae, and Magnaporthaceae. Most of the genera that showed *in silico* amplification with the selected primer set belonged mainly to Magnaporthe species, which are phylogenetically very close to Ggt (**Supplementary Figure 2**). To the best of our knowledge, *Manaporthiopsis panicorum*, *Colletotrichum* sp., and *Nakataea oryzae* have been identified as root pathogenic fungi of grass, bamboo, and rice, respectively (**Supplementary Table 2**).

To determine the specificity of the selected primer set, a PCR reaction was performed using DNA extracted from nine different fungal species used as negative controls. The obtained results showed only positive amplifications in the samples containing Ggt (**Supplementary Figure 3**).

### Primer Validation Using Real-Time PCR and Detection of Ggt in Wheat Roots

Real-time PCR was performed to evaluate the amplification using the selected primer set on DNA from Ggt. Standard regression lines were generated for *C*_T_ values using genomic DNA ranging from 0.8 to 8 × 10^-5^ ng μL^-1^. The regression equation for the DNA standard curve was *y* = -3.337*x* + 42.354, with *r^2^* = 0.98, efficiency of 98%, and a detection limit of 0.08 pg μL^-1^ (**Supplementary Figures 4A,B**). The dissociation curves indicated the presence of a single amplicon (**Supplementary Figure 4C**). The specificity of the primer set was confirmed after identification of the amplicons as *Gaeumannomyces graminis* (Sacc.) by sequencing (accession numbers: **Supplementary Figure 4D**).

**FIGURE 6:**
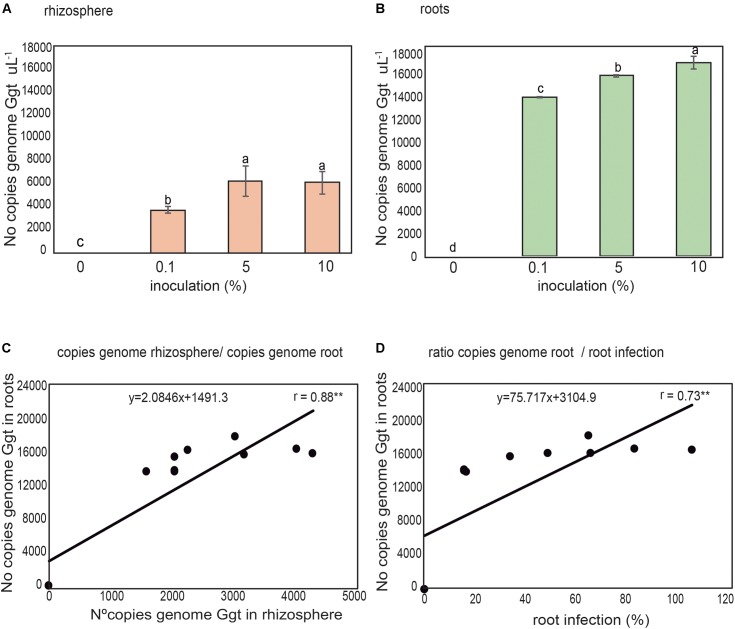
Detection and quantification of number of Ggt genome copies in rhizospheric soil **(A)** and wheat roots **(B)** 40 days after inoculation with 0, 0.1, 5, and 10% of Ggt inoculum. Relation between rhizosphere soil and root Ggt genome copies **(C),** and ratio between Ggt genome copies in the roots and root infection **(D)**. Tukey test to compare treatments means, values followed by the same letter do not differ at *P* < 0.05 (*n* = 3). ^∗^Represents statistically significant correlation (*P* < 0.05), ^∗∗^represent statistically significant correlation (*P* < 0.01).

**FIGURE 7:**
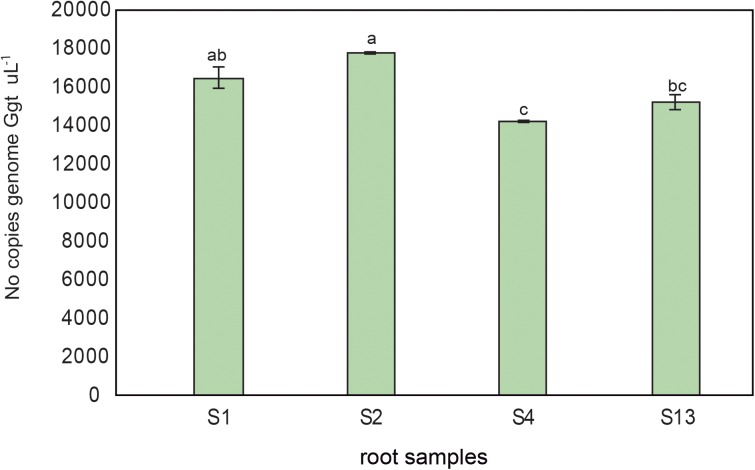
Ggt DNA quantification by quantitative PCR (number of copies genome Ggt uL^-1^) in roots from wheat plants growing in conducive soil 1 (S1), or suppressive soils 2, 4, and 13 (S2, S4, and S13). Tukey test to compare treatments means, values followed by the same letter do not differ at *P* < 0.05 (*n* = 3).

The Ggt DNA present in rhizosphere soils and wheat roots inoculated with increasing concentrations of Ggt inoculum (0, 0.1, 5, and 10% respect of total volume of soils) was quantified by real-time PCR to validate the selected primer set. The results revealed that the concentrations of Ggt in the rhizosphere soils (**Figure 6A**) were lower than in infected roots (**Figure 6B**). However, the concentration of Ggt DNA in the rhizosphere was directly related to the concentration of Ggt DNA in the root (*y* = 0.3706*x*–0.0485, *r* = 0.88, **Figure 6C**). Likewise, we also found that the progress of the take-all disease, evidenced by the blackening of the wheat roots, was directly correlated with the increase in Ggt DNA concentration in the roots samples, with *Y* = 0.0473 + 1.9281, *r* = 0.73, (**Figure 6D**). Due to high specificity of primer set designed we determined Ggt DNA concentration in wheat roots from Experiment 1, growing in suppressive soils (2, 4, and 13) or conducive soil (soil 1). As shown in **Figure 7**, only roots grown in soils 4 and 13 display significantly less Ggt than those growing in the conducive control. In fact, roots from soil 2 show similar Ggt concentration than those in the conducive control soil (around 16500 copies Ggt genome uL^-1^).

### Isolation of Endophytic Bacteria From Wheat Grown in Suppressive Soils and Antagonistic Activity Agains Ggt

Due the importance of endophytic bacteria denoted in this study, we isolated nine endophytic bacteria from suppressive soils: ES2-1, ES2-2, and ES2-3 from soil 2; ES4-1, ES4-2, and ES4-3 from soil 4 and ES13-1, ES13-2, and ES13-3 from suppressive soil 13. The isolated strains were then compared in their ability to inhibit Ggt growth *in vitro*. Only strains ES2-2, ES2-3, and ES13-3 inhibited mycelia growth 7 days after inoculation (**Table 4**). The fungal inhibition was around 15% for ES2-2, 20% for ES2-3 and 20% for ES13-3. The rest of the strains did not show fungal inhibitory activities as compared to the control. The identification and phylogenetic affiliation of these isolates based on 16S rRNA gene partial sequencing is revealed that the endophytic bacteria with antagonistic activity were species from the genera *Serratia* (ES2-3, ES13-3) and *Enterobacter* (ES2-2, **Table 5**).

**Table 4:** Inhibition of *Gaeumannomyces graminis* var. *tritici* growth by selected endophytic strains (ES) isolated from suppressive soils (3 isolated strains per each soil) 3, 5, and 7 days after incubation.

Strain	Diameters of fungal inhibition (cm)		
	**Day 3**	**Day 5**	**Day 7**
Ggt Control	0.23 ± 0.0^a^	1.36 ± 0.01^a^	2.07 ± 0.05^ab^
ES2-1	0.23 ± 0.02^a^	1.03 ± 0.03^c^	1.83 ± 0.04^bcd^
E2-2	0.18 ± 0.02^abc^	1.05 ± 0.04^c^	**1.76 ± 0.09^*cd*^**
ES2-3	0.22 ± 0.02^a^	1.22 ± 0.03^b^	**1.71 ± 0.08^*cd*^**
ES4-1	0.17 ± 0.02^abc^	1.31 ± 0.03^ab^	2.14 ± 0.03^a^
ES4-2	0.20 ± 0.02^ab^	1.30 ± 0.03^ab^	1.98 ± 0.07^abc^
ES4-3	0.14 ± 0.01^bc^	1.25 ± 0.02^ab^	1.88 ± 0.04^abcd^
ES13-1	0.11 ± 0.02^c^	1.09 ± 0.02^c^	1.88 ± 0.04^abcd^
ES13-2	0.13 ± 0.01^c^	1.09 ± 0.02^c^	1.85 ± 0.05^abcd^
ES13-3	0.13 ± 0.01^c^	1.02 ± 0.03^c^	**1.67 ± 0.10^*e*^**


*Data in the same column not sharing a letter in common are significantly different according to Tukey test, *p* < 0.05. Bold letters denote significant differences of fungal growth respect to control.*

**Table 5:** Phylogenetic affiliation of endophytic bacteria isolated from suppressive soils with antagonistic activity.

Isolate	Closest relatives or cloned sequences (accession no.)	Similarity	Accession N°
*Enterobacter* sp(ES2-2)	*Enterobacter* sp. PGPR bacteria with ACCd production capacity (KM250113.1)	99%	MF623051
*Serratia* sp(ES2-3)	*Serratia* sp. Biocontrol of corn root worms in mayze (E302857.1)	99%	MF623052
*Serratia* sp(ES13-3)	*Serratia* sp. PGPR bacteria with biocontrol capacity (KX373960.1)	98%	MF623050


## Discussion

The important role of microbial communities on take-all suppression has been reported by several studies (Mazzola, 2002; Siddiqui and Shaukat, 2002; Löbmann et al., 2016; Durán et al., 2017). A recent study showed that extensive wheat cropping (monoculture), practiced by small Mapuche communities, plays an essential role in the presence of microorganisms involved in soil suppression, identifying six suppressive soils (Durán et al., 2017). Here, we followed up in the study of three of these suppressive soils to get further insights in the structure of the microbial community (total bacteria, actinomycete, total fungi, and ascomycete) and the mechanisms involved in disease suppression. With this aim, we also designed specific primers to quantify Ggt in soil and plant tissues to evaluate the relationship between disease suppression and Ggt abundance.

Remarkably, in this study we found that the total endophytic bacteria communities were similar in all suppressive soils (**Figure 1A**), clearly differentiated from those in the conductive control soil (soil 1). Therefore, we hypothesize that endophytic bacteria have an important role in take-all disease suppression. Additionally, we found that the ascomycete group (to which the pathogen belongs) in suppressive soils are associated with endophytic bacteria in comparison with the rest of the microbial groups analyzed (**Figure 4**), confirming the hypothesis that bacteria could be linked to a specific form of suppression, as proposed earlier by Baker and Cook (1974). Later, Coombs et al. (2004) shown the importance of some strains of actinobacteria in reducing Ggt disease symptoms up to 70%, under steamed soil and field conditions. A similar role of actinomycetes groups was found in suppression of other soilborne pathogens as *Pythium*, *Phytophthora, Ustilago crameri, Rhizoctonia solani*, *Fusarium oxysporum*, and *Ralstonia solani* (El-Tarabily et al., 1997; Latz et al., 2016; van der Voort et al., 2016; Han et al., 2017; Trivedi et al., 2017). However, in this study we found no evidence of a potential role of actinomycetes neither in total fungal community structure in Ggt suppression. Regarding rhizosphere microorganisms, we found that their community structure was more related to essential parameters of volcanic-ash-derived soils as pH, OM and Al Sat (**Figure 3**) than to soil suppression. Thus, biodiversity of the rhizosphere suppressive soils was higher when pH and SOM were high, and lower in relation to Al Sat, as we reported previously (Durán et al., 2017). Through DGGE analyses of the microbial communities other authors showed similar banding patterns in suppressive and conducive soils, supporting that rhizosphere communities have no direct influence over soil suppressive effects (Chng et al., 2015).

The study of mechanisms involved in disease suppression require accurate pathogen quantification methods. To evaluate the abundance of Ggt in suppressive soils we designed an optimized primer set for quantification of *G. graminis* var. *tritici* (Ggt) in rhizosphere and infected roots based on the internal transcribed spacer ITS2 ribosomal region. Previous reported primers (Bithell et al., 2012; Keenan et al., 2015) were not specific according to a similarity search using Primer-BLAST and PCR-based test (**Supplementary Figure 1**). From seven hundred primer combinations able to amplify a fragment of the ITS gene of *G. graminis* KY689233, we selected GGT2F and GGT168R because of their high efficiency (98%) and specificity. The detection limit was 24 pg of DNA g^-1^ of sample (rhizosphere soil or root); this value is within the range shown in the literature considering that values of 6 pg Ggt DNA g^-1^ or lower present no risk of developing take-all, while values above 80 pg DNA g^-1^ indicate a high risk (Bithell et al., 2012). Thus, our study provide an useful tool for take all disease assessment and specific pathogen quantification.

Interestingly, differences in fungal concentration between suppressive and conducive soils were not consistently found in the present study. Although two out of the three soils showed lower Ggt contents, one of them showed even higher levels of pathogen in the roots than those in conducive soils, confirming that suppressive soils can lead to low disease incidence despite high Ggt concentration. Indeed, the suppressive soil 2 showed more than 16,000 N° copies genome Ggt uL^-1^ on wheat roots, and less than 3% blackening roots, in contrast to plants growing in the conducive control soil, with a similar number of Ggt genome copies but more than 30% blackening roots (Durán et al., 2017). Other studies on suppressive soils have found similar results, with low disease severity despite high Ggt concentrations in roots (Chng et al., 2015). Therefore, we suggest that not only Ggt amount, but its pathogenicity varies in soil, and this natural suppression could be attributed to endophytic bacteria that have co-evolved in these plant-soil system and may affect Ggt virulence or the efficiency of the plant defense mechanisms.

To evaluate the role of culturable bacteria in disease suppression, we isolated endophytic bacteria from roots of wheat grown in suppressive soils. Among them, *Serratia* and *Enterobacter spp* were able to moderately inhibit Ggt mycelia growth (only around 20%) under *in vitro* conditions. Remarkably, previous studies have shown that endophytic *Acinetobacter* sp., *Bacillus* sp. and *Klebsiella* sp. inhibited *G. graminis* mycelia growth *in vitro* from 30 to up to 100% (Durán et al., 2014). These results do not support a specific genera as a major driver of Ggt suppressiveness. Similarly, although early studies attributed Ggt suppression to the presence of *Pseudomonas* 2.4 DAPG producers (Raaijmakers et al., 1997), we only detected 2,4- DAPG-producing bacteria in one out of six suppressive soils analyzed recently (Durán et al., 2017). Therefore, we hypothesize that the suppressiveness against Ggt is not exclusively related to the effect of particular specific antagonistic microorganisms or the presence of different genera interacting at different states of the pathogen infection (Gomez-Expósito et al., 2017; Mhlongo et al., 2018).

Our study represents a step forward in understanding natural disease suppression by providing useful tools in the quantification of the pathogen in soil and plant tissues, and by pointing to a relevant role of endophytic bacteria in Ggt suppressiveness. The use of real-time PCR for early Ggt detection in rhizosphere soils and plants is an excellent tool to predict disease incidence and to guide best agronomic practices to combat the most important fungi affecting wheat worldwide. Our results also suggest the importance of the potential activation or fortification of plant defense mechanisms in disease suppression, opening new research lines beyond the identification of antagonistic microorganisms. Future studies should explore plant defense mechanisms in conducive and suppressive soils and should also consider next- generation sequencing to identify functional endophytic bacteria in Ggt suppression. Research should also aim to understand plant-microbiome coevolution from conducive to suppressive soils in order to contribute to the new concept known as “Know before you Sow” to improve productivity and increase food security worldwide.

## Conclusion

Here we provide a primer set (GGT2F and GGT168R) that is highly specific for the detection and quantification of Ggt in plant tissues and soils as a useful tool for detailed studies on Ggt suppressive soils. Our results point to the endophytic bacteria community from wheat roots grown in suppressive soils as main candidates to be involved in take-all suppression, since their community structure correlated with suppressiveness. In contrast, community structures of rhizosphere microorganisms were more influenced by soil chemical parameters and did not correlate with the suppressive potential of the soils. Remarkably, the lower incidence of take-all disease in suppressive soils did not correlate with a reduced Ggt abundance in wheat roots. Therefore, reduction of the pathogen amount is not necessarily the key factor in suppressiveness. Accordingly, suppressiveness against Ggt could be related with the capacity of endophytic bacteria group more than a direct antagonistic activity. Therefore, microbiome analyses from conducive and suppressive soils from identification and functional points of view are required to identify the endophytic bacteria groups relevant in Ggt suppression.

## Author Contributions

PD wrote the main manuscript text. PD, SV, and PB designed the research. MM, MP, and VC supervised the study. GT and VC analyzed the data. All authors critically revised the manuscript and approved the final version.

## Conflict of Interest Statement

The authors declare that the research was conducted in the absence of any commercial or financial relationships that could be construed as a potential conflict of interest.

## Footnotes

1 http://bioinfo.ut.ee/primer3-0.4.0/

2 https://www.ebi.ac.uk/Tools/msa/clustalo/

## Supplementary Material

FIGURE S1 Unspecific Melt curve of primers: **(A)** GgtEFF1 (5′-CCCTGCAAGCTCTTCCTCTTAG-3′) and GgtEFR1 (5′-GCATGCGAGGTCCCAAAA-3′, Keenan et al., 2015). **(B)** NS5 (5′-AACTTAAAGGAATTGACGGAAG-3′), and GGTRP (5′-TGCAATGGCTTCGTGAA-3′ (Fouly and Wilkinson, 2000).

FIGURE S2 Phylogenetic tree showing the affiliation of Ggt in relation to others strains which primers amplified. The neighbor-joining tree was constructed with representative ITS-2 gene sequences. Bootstrap analysis was performed with 1,000 interactions using uniform rates among sites and same (homogeneous) among lineages.

FIGURE S3 ITS region amplified with GGT2F-GGT168R primer pair tested by conventional polymerase chain reaction against: Lane M, 1-kb-plus ladder marker. Lane 1, *Gaeumannonyces graminis*. Lane 2, inoculum with Ggt (oat). Lane 3, inoculum without Ggt. Lane 4, Rhizosphere without Ggt. Lane 5, Rhizosphere with Ggt, Lane 6, roots without Ggt. Lane 7, roots with Ggt. Lane 8, *Aspergillus niger*. Lane 9, *Mortirella* sp. Lane 10, *Rhizopus* sp. Lane 11, *Thelebolus* sp. Lane 12, *Pseudogymnoascus* sp. Lane 13, *Cosmospora* sp. Lane 14, *Lecanicillium* sp. Lane 15, *Alternaria* sp. Lane 16, *Diaporthe* sp., and Lane 17, Negative control.

FIGURE S4 Standard curve of 10-fold serial dilutions of *Gaeumannomyces graminis* DNA (0.8 × 10^-5^ -0.8 ng μL^-1^) for absolute quantification of genomic DNA generated from pure culture of Ggt **(A)**. Efficiency % **(B)** and dissociation curve **(C)**, Phylogenetic tree showing the affiliation of Ggt (red letter) in relation to amplicons obtained by selected primers from soil (brown letter), rhizosphere (green letter) and wheat roots (green letter) **(D)**. The neighbor-joining tree was constructed with representative ITS-2 gene sequences. Bootstrap analysis was performed with 1,000 runs using uniform rates among sites and same (homogeneous) among lineages. The accession numbers are in parentheses.

TABLE 1 Pearson correlation between chemical parameters and biodiversity index of endosphere and rhizosphere samples. ^∗^Denote significant differences at *p*<0.05. ^∗∗^Denote significative differences at *p* < 0.01.

TABLE 2 Pearson correlation between endophytic ascomycete and different microbial groups of conducive and suppressive soils.
