# Supplementary figures and images for: Microbial Community Composition in Take-All Suppressive Soils
Source: Front Microbiol. 2018 Sep 19;9:2198. doi: 10.3389/fmicb.2018.02198 (PMC6156431; doi:10.3389/fmicb.2018.02198)

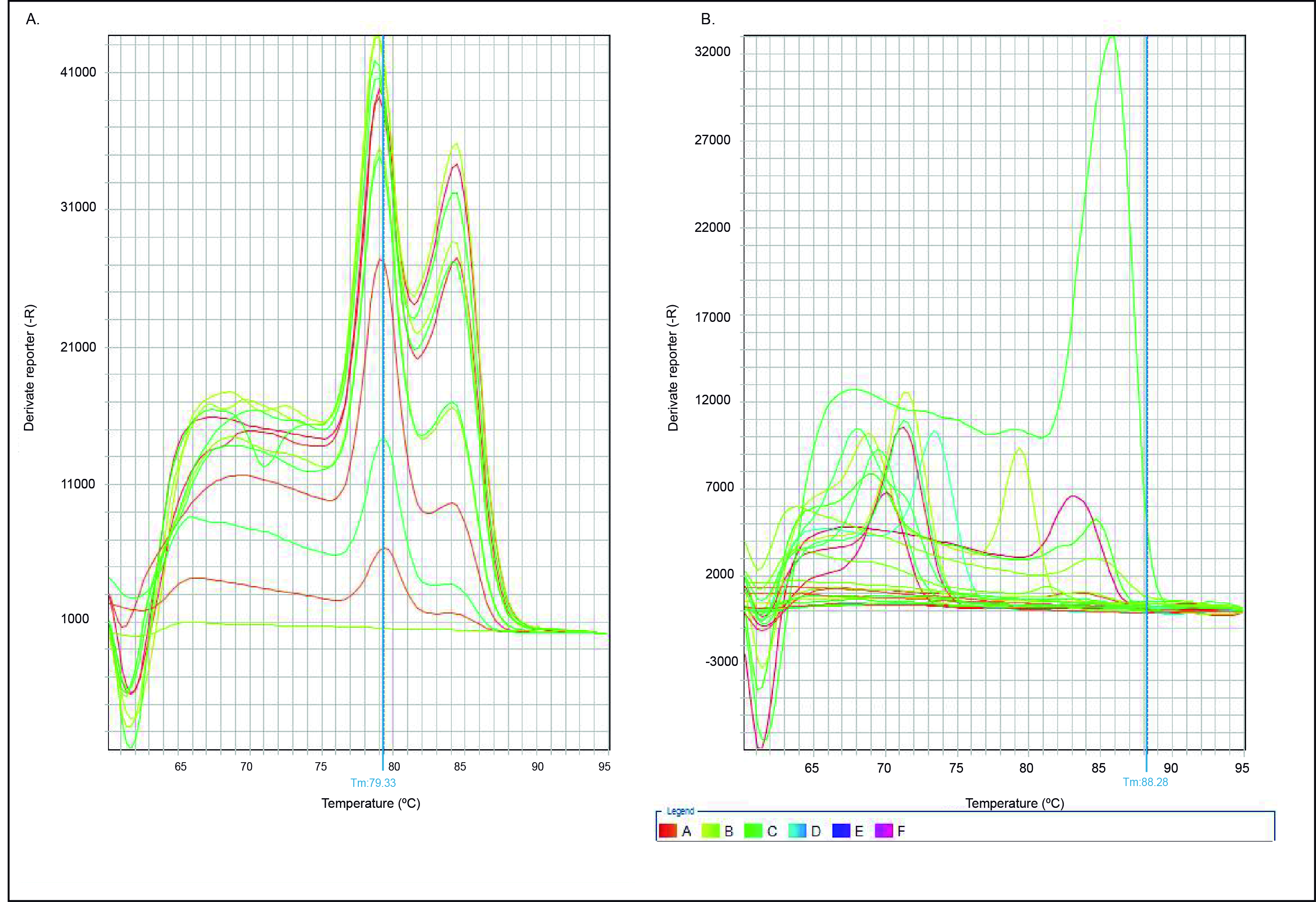

Supplement: FIGURE S1 — Unspecific Melt curve of primers: (A) GgtEFF1 (5′-CCCTGCAAGCTCTTCCTCTTAG-3′) and GgtEFR1 (5′-GCATGCGAGGTCCCAAAA-3′, Keenan et al., 2015). (B) NS5 (5′-AACTTAAAGGAATTGACGGAAG-3′), and GGTRP (5′-TGCAATGGCTTCGTGAA-3′ (Fouly and Wilkinson, 2000). [file Image_1.TIF]

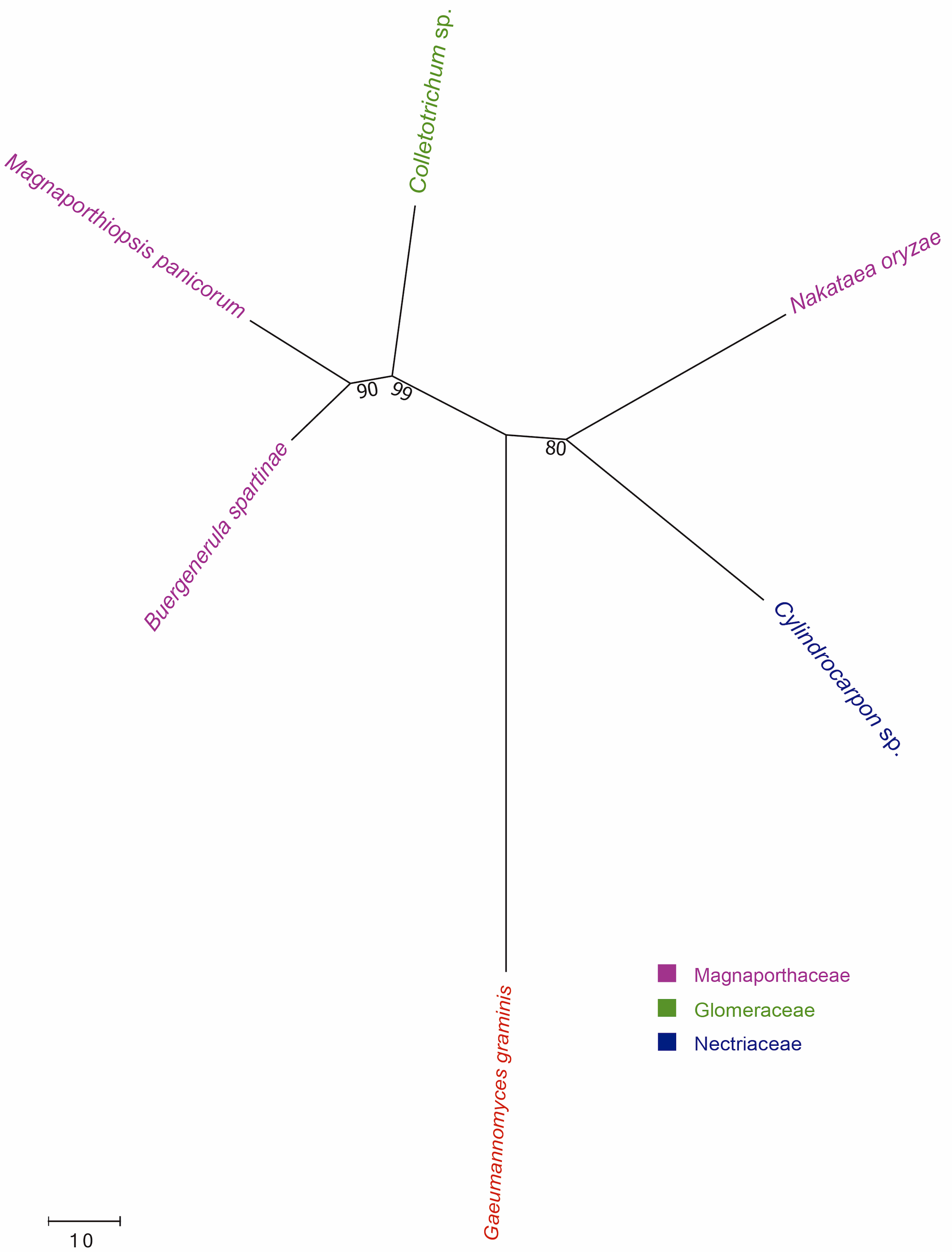

Supplement: FIGURE S2 — Phylogenetic tree showing the affiliation of Ggt in relation to others strains which primers amplified. The neighbor-joining tree was constructed with representative ITS-2 gene sequences. Bootstrap analysis was performed with 1,000 interactions using uniform rates among sites and same (homogeneous) among lineages. [file Image_2.TIF]

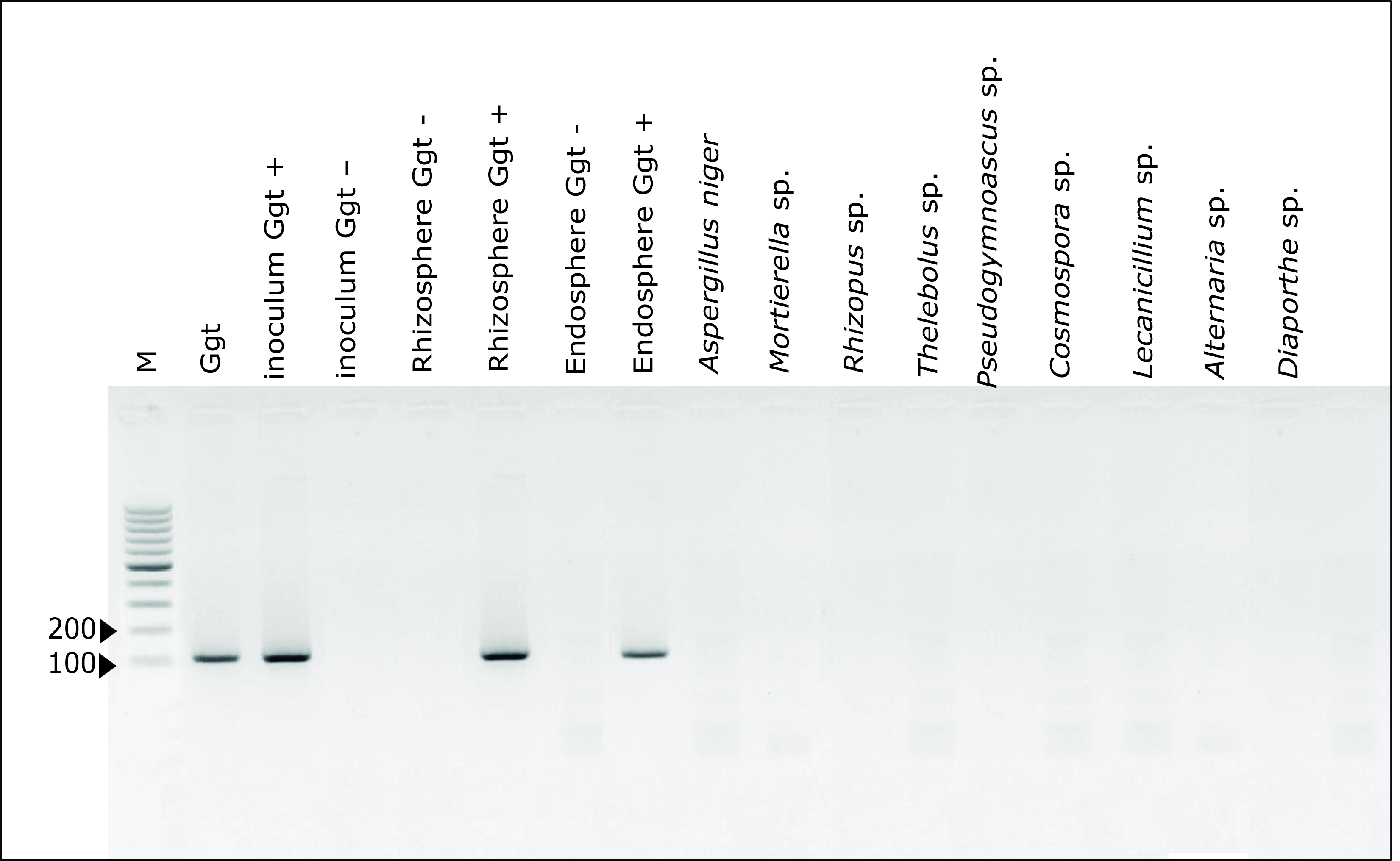

Supplement: FIGURE S3 — ITS region amplified with GGT2F-GGT168R primer pair tested by conventional polymerase chain reaction against: Lane M, 1-kb-plus ladder marker. Lane 1, Gaeumannonyces graminis. Lane 2, inoculum with Ggt (oat). Lane 3, inoculum without Ggt. Lane 4, Rhizosphere without Ggt. Lane 5, Rhizosphere with Ggt, Lane 6, roots without Ggt. Lane 7, roots with Ggt. Lane 8, Aspergillus niger. Lane 9, Mortirella sp. Lane 10, Rhizopus sp. Lane 11, Thelebolus sp. Lane 12, Pseudogymnoascus sp. Lane 13, Cosmospora sp. Lane 14, Lecanicillium sp. Lane 15, Alternaria sp. Lane 16, Diaporthe sp., and Lane 17, Negative control. [file Image_3.TIF]

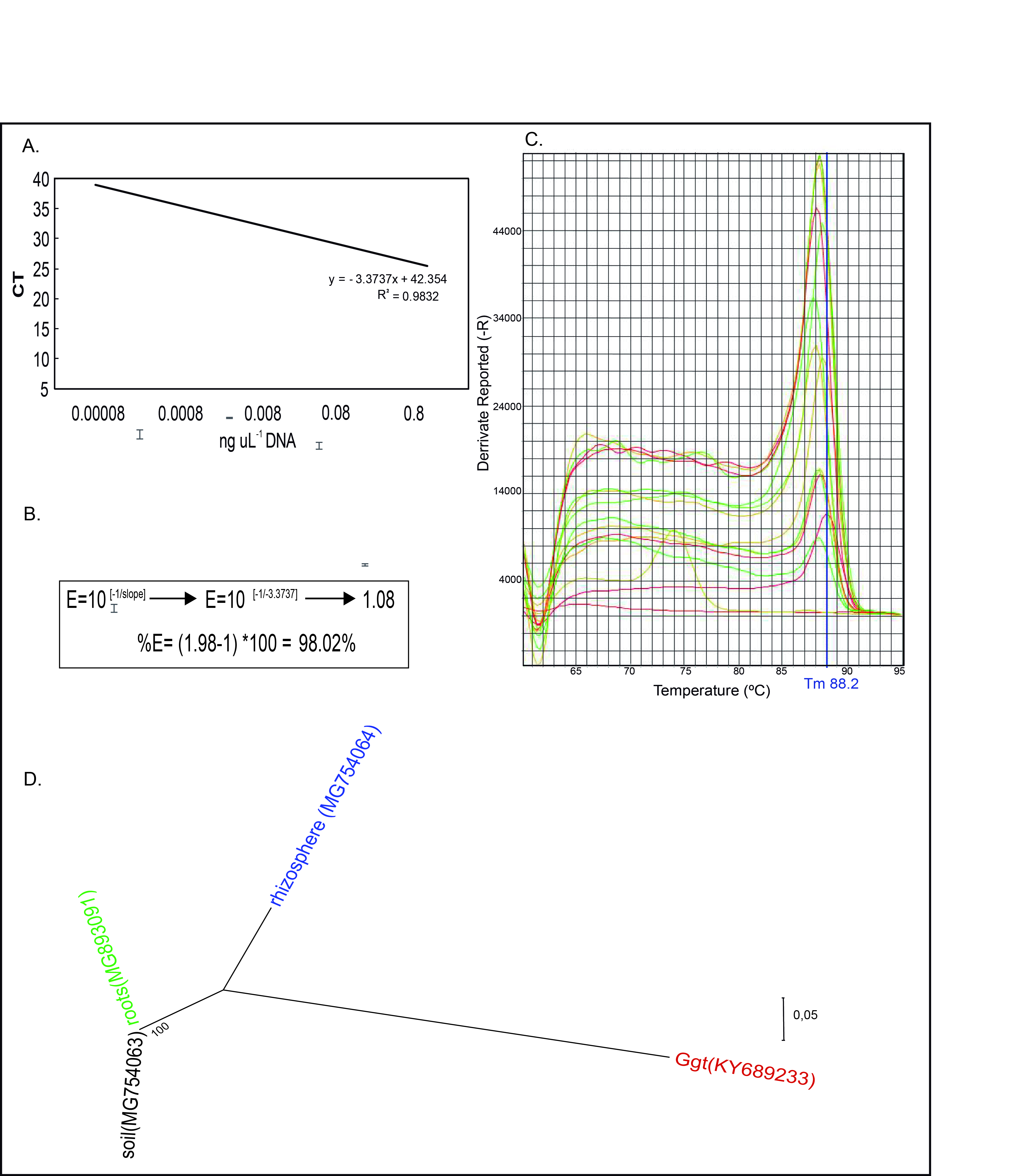

Supplement: FIGURE S4 — Standard curve of 10-fold serial dilutions of Gaeumannomyces graminis DNA (0.8 × 10^-5^ -0.8 ng μL-1) for absolute quantification of genomic DNA generated from pure culture of Ggt (A). Efficiency % (B) and dissociation curve (C), Phylogenetic tree showing the affiliation of Ggt (red letter) in relation to amplicons obtained by selected primers from soil (brown letter), rhizosphere (green letter) and wheat roots (green letter) (D). The neighbor-joining tree was constructed with representative ITS-2 gene sequences. Bootstrap analysis was performed with 1,000 runs using uniform rates among sites and same (homogeneous) among lineages. The accession numbers are in parentheses. [file Image_4.JPEG]
