# Supplementary material for: Microbial Community Composition in Take-All Suppressive Soils
Source: Front Microbiol. 2018 Sep 19;9:2198. doi: 10.3389/fmicb.2018.02198 (PMC6156431; doi:10.3389/fmicb.2018.02198)
Supplement: TABLE 1 — Pearson correlation between chemical parameters and biodiversity index of endosphere and rhizosphere samples. ∗Denote significant differences at p<0.05. ∗∗Denote significative differences at p < 0.01. [file Table_1.DOC]

**Supplementary Table 1:** Pearson correlation between chemical parameters and biodiversity index of endosphere and rhizosphere samples

* denote significant differences at p<0.05

** denote significative differences at p<0.01

**A) Total bacteria**

*Endosphere* rhizosphere

|  | P | K | pH | MO | Sat Al | CICE | S. Bases |  |  |  | P | K | pH | OM | Sat Al | CICE | S. Bases |
| --- | --- | --- | --- | --- | --- | --- | --- | --- | --- | --- | --- | --- | --- | --- | --- | --- | --- |
|  |  |  |  |  |  |  |  |  |  |  |  |  |  |  |  |  |  |
| S | 0.48 | 0.34 | -0.41 | **-0.61*** | **0.68*** | -0.24 | -0.32 |  |  | S | 0.13 | 0.12 | **0.84**** | **0.75**** | **-0.90**** | 0.31 | 0.40 |
| N | 0.39 | 0.54 | -0.25 | **-0.61*** | 0.47 | 0.07 | 0.00 |  |  | N | 0.07 | 0.27 | **0.64*** | **0.45** | **-0.73**** | 0.44 | 0.50 |
| d | 0.48 | 0.27 | -0.42 | -0.58 | **0.70*** | -0.31 | -0.38 |  |  | d | 0.13 | 0.10 | **0.85**** | **0.76**** | **-0.90**** | 0.29 | 0.40 |
| J' | 0.58 | 0.60 | -0.14 | **-0**.**64*** | 0.51 | 0.00 | -0,07 |  |  | J' | -0.31 | -0.04 | -0.26 | -0.14 | 0.09 | 0.16 | 0.13 |
| H' | 0.54 | 0.42 | -0.33 | **-0**.**62*** | **0**.**65*** | -0.20 | -0,28 |  |  | H' | 0.04 | 0.12 | **0**.**74**** | **0**.**68*** | **-0**.**84**** | 0.34 | 0.43 |
| 1- λ | 0.59 | 0.53 | -0.23 | **-0**.**63*** | 0.59 | -0.12 | -0,19 |  |  | 1- λ | -0.04 | 0.06 | **0.60*** | **0.61*** | **-0.75**** | 0.32 | 0.40 |

**B. Actinomycetes**

*Endosphere* rhizosphere

|  | P | K | pH | MO | Sat Al | CICE | S. Bases |  |  |  | P | K | pH | OM | Sat Al | CICE | S. Bases |
| --- | --- | --- | --- | --- | --- | --- | --- | --- | --- | --- | --- | --- | --- | --- | --- | --- | --- |
| S | -0.10 | -0.46 | -0.27 | 0.14 | 0.26 | -0.46 | -0.46 |  |  | S | -0.39 | **-0.94**** | -0.46 | 0.45 | 0.14 | **-0.65*** | **-0.62*** |
| N | 0.13 | 0.06 | -0.07 | -0.09 | 0.11 | -0.09 | -0.10 |  |  | N | 0.32 | -0.49 | -0.39 | 0.22 | 0.38 | **-0.81**** | **-0.80**** |
| d | -0.14 | -0.52 | -0.28 | 0.18 | 0.26 | -0.47 | -0.47 |  |  | d | -0.43 | **-0.94**** | -0.46 | 0.45 | 0.12 | **-0.61*** | **-0.59*** |
| J' | -0.03 | 0.01 | 0.07 | -0.03 | -0.13 | 0.05 | 0.06 |  |  | J' | 0.38 | -0.08 | -0.41 | -0.28 | **0.59*** | -0.54 | **-0.58*** |
| H' | -0.13 | -0.45 | -0.23 | 0.15 | 0.19 | -0.49 | -0.40 |  |  | H' | -0.26 | **-0.88**** | -0.54 | 0.31 | 0.30 | **-0.73**** | **-0.72**** |
| 1- λ | -0.06 | -0.29 | -0.15 | 0.09 | 0.13 | -0.29 | -0.28 |  |  | 1- λ | 0.00 | 0.07 | 0.32 | 0.34 | 0.01 | 0.01 | 000 |

**C. Total fungi**

*Endosphere rhizosphere*

|  | P | K | pH | MO | Sat Al | CICE | S. Bases |  |  |  | P | K | pH | OM | Sat Al | CICE | S. Bases |
| --- | --- | --- | --- | --- | --- | --- | --- | --- | --- | --- | --- | --- | --- | --- | --- | --- | --- |
| S | -0.10 | -0.46 | -0.27 | 0.14 | 0.26 | -0.46 | -0.46 |  |  | S |  | 0.22 | 0.55 | **0.72**** | 0.22 | **-0.71**** | 0.57 |
| N | 0.13 | 0.06 | -0.07 | -0.09 | 0.11 | -0.09 | -0.10 |  |  | N |  | 0.30 | 0.50 | **0.65*** | 0.20 | **-0.62*** | 0.44 |
| d | -0.14 | -0.52 | -0.28 | 0.18 | 0.26 | -0.47 | -0.47 |  |  | d |  | 0.19 | 0.54 | **0.72**** | 0.22 | **-0.72**** | **0.59*** |
| J' | -0.03 | 0.01 | 0.07 | -0.03 | -0.13 | 0.05 | 0.06 |  |  | J' |  | 0.25 | -0.21 | **-0.73**** | -0.47 | **0.86**** | **-0.66*** |
| H' | -0.13 | -0.45 | -0.23 | 0.15 | 0.19 | -0.40 | -0.10 |  |  | H' |  | 0.11 | 0.45 | **0.62*** | 0.20 | **-0.66*** | 0.55 |
| 1- λ | -0.06 | -0.29 | -0.15 | 0.09 | 0.13 | -0.29 | -0.28 |  |  | 1- λ |  | -0.01 | 0.40 | 0.48 | 0.08 | -0.53 | 0.54 |

**D. Ascomycete**

*Endosphere rhizosphere*

|  | P | K | pH | MO | Sat Al | CICE | S. Bases |  |  |  | P | K | pH | OM | Sat Al | CICE | S. Bases |
| --- | --- | --- | --- | --- | --- | --- | --- | --- | --- | --- | --- | --- | --- | --- | --- | --- | --- |
| S | 0.47 | **0.88**** | 0.20 | **-0.67*** | 0.17 | 0.44 | 0.39 |  |  | S | 0.07 | -0.29 | **-0.71*** | -0.44 | **0.89**** | **-0.60*** | **-0.68*** |
| N | 0.33 | **0.88**** | 0.30 | -0.46 | -0.08 | 0.59 | 0.57 |  |  | N | 0.29 | 0.23 | -0.21 | -0.50 | 0.54 | -0.16 | -0.23 |
| d | 0.48 | **0.86**** | 0.17 | **-0.68*** | 0.21 | 0.40 | 0.34 |  |  | d | 0.03 | -0.37 | **-0.74**** | -0.38 | **0.88**** | **-0.64*** | **-0.71**** |
| J' | 0.57 | -0,03 | -0.08 | -0.07 | 0.37 | -0.58 | -0.57 |  |  | J' | **0.62*** | 0.11 | 0.09 | -0.02 | 0.19 | -0.41 | -0.41 |
| H' | 0.53 | **0.81**** | 0.08 | **-0.70*** | 0.30 | 0.28 | 0.22 |  |  | H' | 0.46 | -0.11 | -0.31 | -0.23 | **0.61*** | **-0.62*** | **-0.66*** |
| 1- λ | 0.67 | **0.71**** | 0.02 | **-0.67*** | 0.39 | 0.10 | 0.04 |  |  | 1- λ | 0.51 | -0.03 | -0.14 | -0.14 | 0.43 | -0.53 | -0.55 |
