# Supplementary material for: Microbial Community Composition in Take-All Suppressive Soils
Source: Front Microbiol. 2018 Sep 19;9:2198. doi: 10.3389/fmicb.2018.02198 (PMC6156431; doi:10.3389/fmicb.2018.02198)
Supplement: TABLE 2 — Pearson correlation between endophytic ascomycete and different microbial groups of conducive and suppressive soils. [file Table_2.DOCX]

**Supplementary Table 2.** Pearson correlation between endophytic ascomycete and different microbial groups of conducive and suppressive soils

| *Conducive soil* | |  |  |  |  |  |
| --- | --- | --- | --- | --- | --- | --- |
|  | Bac-endo | Bac-rhizo | Actino-endo | Actino-rhizo | Fungi-endo | Fungi- rhizo |
| Asco-endo | -0.97 | -0.76 | 0.73 | 0.92 | 0.99 | -0.97 |
| Asco- rhizo | 0.59 | 0.18 | **-0.99*** | -0.47 | -0.66 | 0.61 |
|  |  |  |  |  |  |  |
| *Suppressive soil* | |  |  |  |  |  |
|  | Bac-endo | Bac-rhizo | Actino-endo | Actino-rhizo | Fungi-endo | Fungi- rhizo |
| Asco-endo | **0.80*** | **-0.74*** | **0.74*** | 0.14 | -0.05 | -0.17 |
| Asco-rhizo | **0.73*** | -0.53 | 0.37 | -0.04 | -0.06 | -0.53 |
